# COVID-19: Risk of increase in smoking rates among England’s 6 million smokers and relapse among England’s 11 million ex-smokers

**DOI:** 10.3399/bjgpopen20X101067

**Published:** 2020-04-08

**Authors:** Pooja Patwardhan

**Affiliations:** 1 Sessional GP, and Medical Director, Centre for Health Research and Education, UK

**Keywords:** Smoking relapse, COVID-19, Smoking cessation, coronavirus, general practice, primary healthcare

## ​Possible unintended consequences of social isolation and mental stress

As a practising GP, I am concerned about those who might get seriously unwell due to the respiratory virus SARS-CoV-2. As a member of the Royal College of General Practitioners (RCGP), I join hands with the public health and other healthcare communities to focus on acute measures in this time of national emergency to save lives from COVID-19. At the same time, I am also worried about those who may not currently be in the high-risk group for COVID-19 but will be self-isolating and maintaining social distance. As the world goes into lockdown, social distancing and self-isolation are likely to make the society very lonely and life more stressful.

We are already seeing patients in general practice who are understandably stressed in these extra-ordinary circumstances. With my experience of working in preventive medicine and smoking cessation, the uncertainty and the stress might push current smokers to smoke more cigarettes and ex-smokers to relapse back to smoking.

## A perfect (bad) storm for relapsing and smoking more

There are many people really worried about the health of their ageing parents whom they cannot now visit, about the financial and health impact COVID-19 will have on their lives, and about all the other uncertainties around it. Staying at home, people are listening to all sorts of news — some true, some rumours — about what the future might have in store for them. The feeling of helplessness will be pronounced when the usual comfort of sharing their worries and the stresses of life with friends and extended family are not available anymore. As a consequence, we can expect the stress levels for everyone in the population to increase dramatically. The COVID-19 pandemic is bound to negatively impact the mental health of the population. Chances of conditions like anxiety and depression getting worse are high. Stress and worsening mental health are known predisposing factors for increased smoking (quantity and frequency) as well as relapse.^1^

There may be people who have cut down smoking as they are not allowed to smoke in their workplace. There will be ex-smokers who have successfully quit smoking by going to the gym or joining a local sports group to help them. There may be some, who have stopping smoking by taking up a new job or starting a new business. These — and so many other ways that people may have used to shift towards a healthier lifestyle — may suddenly be unavailable and inaccessible in their lives.

Unfortunately, with the isolation policy, any local face to face stop smoking support will not be available to people who are currently in the middle of their quitting journey.

## Decades of tobacco control successes at risk of reversing

The UK has always been on the forefront of national level tobacco control policies and guidelines and has achieved great successes with smoking cessation campaigns. This has resulted in England having over 11 million ex-smokers.^2^ England’s 2018 adult smoking prevalence of 14.4%^3^ was a result of successful tobacco control campaigns and offering smoking cessation support to smokers across the country. However, the number of people setting a quit date fell for the sixth consecutive year in 2017/18, to 274 021. This represented a decrease of 11% on 2016/17. In 2017/18, the number of successful self-reported quitters also fell for the fifth consecutive year, to 138 426. This was a decrease of 11% on 2016/17.^3^ It is evident that England is on a final but difficult stretch of achieving smoking cessation among the remaining 6.1 million smokers.^4^

With this background, an increase in relapse to smoking among the 11 million ex-smokers as well as increased smoking rates in current smokers will set back England’s smokefree ambitions by years, and possibly decades. COVID-19 threatens to do exactly that. We need to act now to prevent another public health calamity as COVID-19 rages on.

Along with the long-term risks, smoking will also increase people’s risk of acute respiratory diseases, including risk of serious symptoms from COVID-19.^5^ This, in turn, will significantly add burden to the already overstretched healthcare system.

## Helping current and ex-smokers stay away from cigarettes in these challenging times

I would urge the UK government, NHS, and Public Health England to include advice about pre-empting and managing the risk of relapse as well as increased urge to smoke more in their documents regarding ‘Coronavirus and what people need to do: advice on social distancing and isolation’. People need to be made aware that they might be at a risk of relapsing back to smoking in the current stressful environment. Similar written advice should be included on all GP surgeries’ websites.

In most places now, doctors and nurses, especially those working in primary care, are providing care to non-COVID patients by telephone, electronic, or in-person consultations. I would strongly recommend that they proactively look for ‘smoker’ or ‘ex-smoker’ status on every patient’s medical records system. They should offer opportunistic advice to all these patients on relapse prevention and to watch for increased smoking tendency. Even with the time constraints, they can direct people to the detailed written advice on their website. The short-term as well as long-term benefits of this will be worth the time spent by these professionals.

In addition, all GP surgeries can send a text message/ email to all patients known to be smokers or ex-smokers directing them to this advice.

Recommendations for the relapse prevention advice incude:

- Signposting to NHS Smokefree website (NHS Smokefree) along with the details of National Smokefree telephone lines and Smokefree apps.^6^
- Advice on pre-empting the risk of going back to/increasing smoking
- Advice on how to fight the new smoking urge early with fast-acting nicotine replacement products (nicotine gums, mouth sprays, and e-cigarettes)
- Up-titrating on nicotine by dual use (combinations of short- and long-acting nicotine replacement therapy and/or e-cigarettes) when needed. People should, if appropriate, keep these cessation aids easily accessible and available to use if they are struggling to control the urge.

A clear message needs to reach all smokers, ex-smokers, and their healthcare advisors: these are understandably stressful times but smoking is not a solution. The RCGP membership and the wider healthcare community need to warn their patients of the likely urge to smoke and offer strategies on staying smokefree with all available cessation aids and resources. It is vital to help ex-smokers maintain their hard-earned quit status and help current smokers cut down to quit, to prevent additional burden on our NHS in the short term and to achieve a smokefree England by 2030.^7^
