# Correction: COVID-19: Risk of increase in smoking rates among England’s 6 million smokers and relapse among England’s 11 million ex-smokers

**DOI:** 10.3399/bjgpopen21X101161

**Published:** 2021-01-19

In the Practice & Policy article by Patwardhan, COVID-19: Risk of increase in smoking rates among England’s 6 million smokers and relapse among England’s 11 million ex-smokers *BJGP Open* 2020; DOI: https://doi.org/10.3399/bjgpopen20X101067, the Competing Interests section stated “Along with practising as a GP, PP is also a paid director for Centre for Health Research and Education (CHRE) UK, which works on projects on smoking cessation globally. PP or CHRE have not received any funding from pharmaceutical, electronic cigarette, or tobacco industries”. This section should have stated: “Along with practising as a GP, PP is also a paid director for Centre for Health Research and Education (CHRE) UK, an independent company, which works on projects on smoking cessation globally. CHRE has received grants from Foundation for a Smoke-free World, Inc (FSFW) for some of its smoking cessation projects. FSFW describes itself as a non-profit, independent organisation which is funded by Philip Morris International Global Services, Inc. For this article, PP did not receive specific funding and it has been written in a personal capacity, to create awareness amongst health professionals on the need for providing smoking cessation support during the COVID-19 pandemic.”

We apologise for this error. The online version has been corrected.

